# Metabolic Differences in Glutamine Utilization Lead to Metabolic Vulnerabilities in Prostate Cancer

**DOI:** 10.1038/s41598-017-16327-z

**Published:** 2017-11-23

**Authors:** Niki Marie Zacharias, Christopher McCullough, Sriram Shanmugavelandy, Jaehyuk Lee, Youngbok Lee, Prasanta Dutta, James McHenry, Linda Nguyen, William Norton, Lawrence W. Jones, Pratip K. Bhattacharya

**Affiliations:** 1 Department of Cancer Systems Imaging, The University of Texas MD Anderson Cancer Center, Houston, TX USA; 2 Department of Urology, The University of Texas MD Anderson Cancer Center, Houston, TX USA; 3 Institute for Bioscience and Biotechnology Research, National Institute of Standards and Technology, Rockville, MD USA; 4 Department of Bionano Technology. Hanyang University, ERICA campus, Ansan, Korea; 5 Department of Veterinary Medicine, The University of Texas MD Anderson Cancer Center, Houston, TX USA; 6 Huntington Medical Research Institutes, Pasadena, CA USA

## Abstract

The new oncologic paradigm of precision medicine is focused on identifying metabolic, proteomic, transcriptomic and genomic variabilities in tumors that can be exploited to tailor treatments and improve patient outcomes. Metabolic changes are a hallmark of cancer, and inhibition of metabolic pathways is now a major strategy in medicinal chemistry for targeting cancers. However, non-invasive biomarkers to categorize metabolic subtypes are in short supply. The purpose of this study was to characterize the intracellular and extracellular metabolic profiles of four prostate cancer cell lines with varying degrees of aggressiveness. We observed metabolic differences between the aggressive prostate cancer cell line PC3 and the even more aggressive, metastatic subline PC3M assessed by hyperpolarized *in vivo* pyruvate studies, nuclear magnetic resonance spectroscopy, and carbon-13 feeding studies. On further examination of the differences between these two cell lines, we found increased glutamine utilization in the metastatic PC3M subline that led directly to sensitivity to glutaminase inhibitor CB-839. Our study supports the theory that metastatic progression increases glutamine utilization and the inhibition of glutaminolysis could have clinical implications.

## Introduction

**Figure 1:**
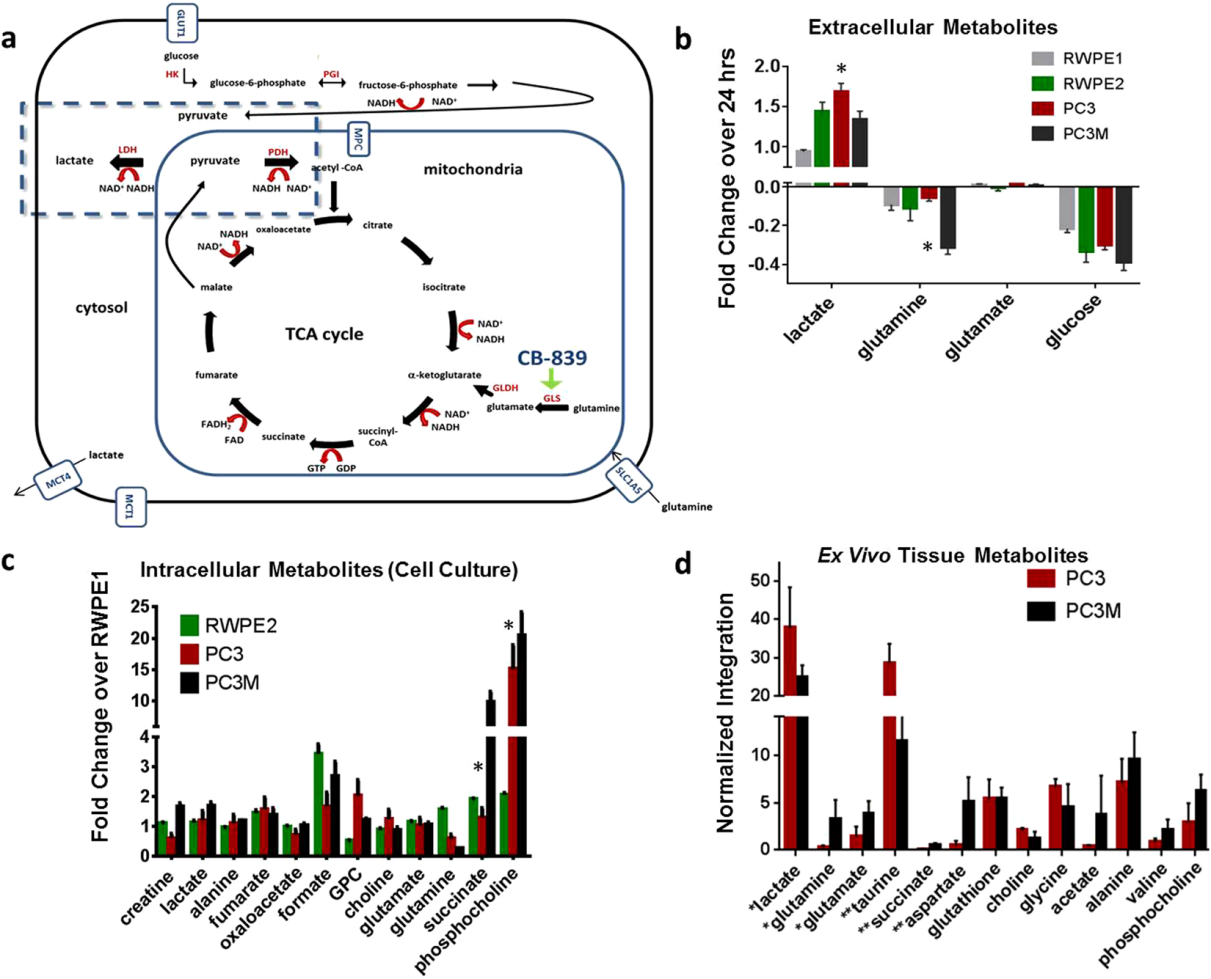
Metabolic differences between PCa cell lines. In all experiments, metabolite values were determined by MRS and values were normalized as stated per experiment. Statistical significance indicated by an asterisk (*). (**a**) In this schematic, partial pathways of glycolysis, glutaminolysis, and the citric acid cycle are shown: red type is specific enzymes, red arrows depict the conversion of cofactors such as NAD^+^, blue type is transporters, and black type is specific metabolites. The dashed box highlights the production of lactate through lactate dehydrogenase (LDH). There are several steps between fructose-6-phosphate and the generation of two equivalences of pyruvate not shown. We were specifically focused on determining the differences in aerobic and anaerobic glycolytic metabolites in PCa cell lines. Abbreviations: HK (hexokinase), PGI (phosphoglucose isomerase), GLDH (glutamate dehydrogenase), GLS (glutaminase), MCT4 and MCT1 (monocarboxylic acid transporters), GLUT1 (glucose transporter), SLC1A5 (glutamine transporter), MPC (mitochondrial pyruvate carrier), MAE (malic enzyme). (**b**) To compare extracellular metabolite changes among cell lines, concentrations of metabolites within a 24-hour period were determined and changes over that period were graphed as shown. Positive bars are metabolites excreted into the media and negative bars are metabolites consumed by the cells over the 24-hour period (n = 5 samples per cell type). Glutamine uptake by PC3M cells was over three fold higher than that by PC3 cells (P < 0.0001). Lactate production by PC3 was 1.3 fold higher than that by PC3M (P < 0.0001). Statistical significance between metabolite levels was determined using two-way ANOVA with Tukey’s multiple comparisons. (**c**) To compare intracellular metabolite levels in cultured cells, metabolite concentrations were determined and the fold change compared to the indolent cell line (RWPE1) was plotted. The levels of metabolites succinate and phosphocholine (PCho) were significantly lower in PC3M cell lysates than in PC3 lysates (P < 0.0004). Statistical significance was determined by two-way ANOVA with Tukey’s multiple comparisons. (**d**) To compare levels of metabolites in *ex vivo tumor* tissues, PC3 and PC3M tumors were excised from mice and samples prepared for analysis (100 mg per sample, PC3 n = 4, PC3M n = 5). Levels of lactate and taurine were significantly higher in PC3 tumors than in PC3M tumors, while levels of aspartate, glutamate, glutamine and succinate were significantly higher in PC3M tumors than in PC3 tumors. Unpaired t-test using Holm-Sidak method was used to determine statistical significance (**P < 0.02, *P < 0.04).

Prostate cancer (PCa) is the second leading cause of cancer death in men in the United States. Despite the approval of multiple new therapies for PCa^1–5^, metastatic disease remains incurable. The metabolism of normal prostate cells versus cancer cells has been revealed in several biochemistry assays to be significantly different^6^. For example, the concentration of citrate is higher in normal prostate than in PCa, reaching concentrations as high as 180 mM in prostatic fluid^7^. The significant reduction in citrate level in PCa^8–10^ results from citrate utilization in other metabolic pathways^6,11^. Several recent publications have found differences between PCa and normal prostate in expression of metabolic enzymes such as glutaminase (GLS)^12,13^, acetyl-CoA synthetase^14^, and the monocarboxylic acid transporters MCT1/MCT4^15–17^ (See Fig. 1a). Our long-term goal is to identify the roles of these metabolites in prostate cancer growth and progression.

The major changes that occur in PCa are similar to what is seen in other cancers including increased glycolysis^18^. The increases in glycolysis in PCa progression has been observed both in cell culture^19^ and *in vivo* using a new metabolic imaging technique called hyperpolarized magnetic resonance^20^. Hyperpolarization can enable a >10,000 fold sensitivity enhancement of the magnetic resonance (MR) signal of contrast agents over Boltzmann polarization^20–23^. More importantly, this signal enhancement is retained on the metabolites of the hyperpolarized molecule allowing direct observation and measurement of metabolic flux and real-time metabolic imaging. The most widely used method for providing hyperpolarized contrast agents for metabolic MR imaging is dynamic nuclear polarization (DNP)^24,25^ and the most studied hyperpolarized ^13^C tracer with DNP is pyruvate. Recently, the first Phase I study with hyperpolarized 1-^13^C pyruvate was completed^26^. Hyperpolarized 1-^13^C pyruvate is taken up by glycolytic cancer cells quickly and is then converted to 1-^13^C lactate through lactate dehydrogenase (LDHA)^20,26–30^. This conversion rate can be measured in real time and *in vivo* by measuring the integration of the lactate signal over the overall hyperpolarized signal (nLac). Multiple laboratories have used hyperpolarized 1-^13^C pyruvate to image the transgenic adenocarcinoma of mouse prostate (TRAMP) model^31^. In the TRAMP model, increases in glycolytic flux were observed in higher histological grade PCa^20^ and was reduced after hormone therapy^32^.

A key nutrient for most cancers is glutamine, and one of the critical steps in its utilization is its conversion to glutamate through the glutaminase enzyme^33–36^. The *GLS* gene encodes two splice variants of glutaminase, kidney-type glutaminase (KGA) and glutaminase C (GAC), collectively called GLS1^37^. The *GLS2* gene encodes two glutaminase isoforms, LGA (liver-type) and GAB, collectively called GLS2^37^. GLS2 isozymes have been shown to be downregulated in several cancers^38^, including acute myeloid leukemia^39^ and breast^40,41^ and lung cancers^42^. In contrast, GLS1 is typically upregulated in cancers^39^. Glutamine can be used not only as a nitrogen source but also as a carbon source and for energy production^33–35^. A recent publication reported that GLS1 was expressed in 68 of 107 (64%) PCa specimens but in only 9 of 37 (24%) benign hyperplastic prostate specimens^12^.

Androgen receptor (AR) signaling is one of the most critical pathways for maintaining prostate growth and normal function; however, AR activation is also important in prostate cancer pathogenesis and progression^43,44^. Androgen ablation is usually a first-line drug with and without resection. However, a subset of patients does not benefit from androgen ablation or in time their cancer evolves and begins to be non-responsive. Androgen insensitive PCa, termed castration resistant, can occur by multiple mechanisms including point mutations in AR gene, generation of splicing variants (e.g. AR-V7), amplification of AR gene, or complete ablation of the AR receptor^43,44^.

We are specifically interested in determining the metabolic differences in castration resistant and metastatic PCa. Our purpose was to further our understanding in the metabolic differences in the extracellular and intracellular metabolic profiles of four PCa cell lines with varying degrees of aggressiveness specifically focusing on glutamine and glucose utilization. Our *in vitro* metabolic results led us to determine the hyperpolarized metabolic flux of 1-^13^C pyruvate in xenograft PC3 and PC3M animal models and the testing of CB-839. CB-839 (Calithera Biosciences) is a nanomolar binder to both major isoforms of glutaminase 1 (GLS1), has good oral bioavailability, and is currently in a Phase I study for solid tumors and leukemia (NCT02071862, NCT02071927)^39,41^.

## Results

### Production of lactate and uptake of glucose and glutamine differ in prostate cancer cells

Changes in the concentrations of extracellular metabolites (consumed and excreted) over 24 hours of growth were measured in four human PCa cell lines: RWPE-1, RWPE-2, PC3, and PC3M^45^. Figure 1 summarizes all of the metabolic profiling data both in cell culture and tumor tissue. Changes observed in selected extracellular metabolites among the four cell lines in 24 hours are shown in Fig. 1b. Positive values represent secreted metabolites, while negative values represent consumed metabolites. Glutamine uptake was over 3 times higher in PC3M cells than in PC3 (P < 0.0001), while lactate production was 1.3 times higher in PC3 cells than in PC3M (P < 0.0001). However, glucose uptake and glutamate utilization is similar in RWPE2, PC3, and PC3M cell lines. Significant differences in intracellular metabolite levels were found between PC3 and PC3M both in cell culture and excised tumor tissues. In cell culture, levels of phosphocholine (PCho) and succinate were significantly different (P < 0.0001) in PC3 and PC3M (Fig. 1c). In addition, intracellular glutamine levels were observed to be lower in the aggressive cell lines (PC3 and PC3M) compared to unaggressive lines but were not statistically significant by two-way ANOVA analysis. The metabolic profile of tumor tissue samples (Fig. 1d), which includes both the cells (intracellular) and interstitial spaces (extracellular), revealed the same differences found in PC3 and PC3M *in vitro* assays: lactate was higher in PC3 tumors than in PC3M tumors, and succinate, aspartate, glutamate and glutamine were higher in PC3M tumors.

### Carbon-13 tracing experiments reveal differences in glutamine utilization

**Figure 2:**
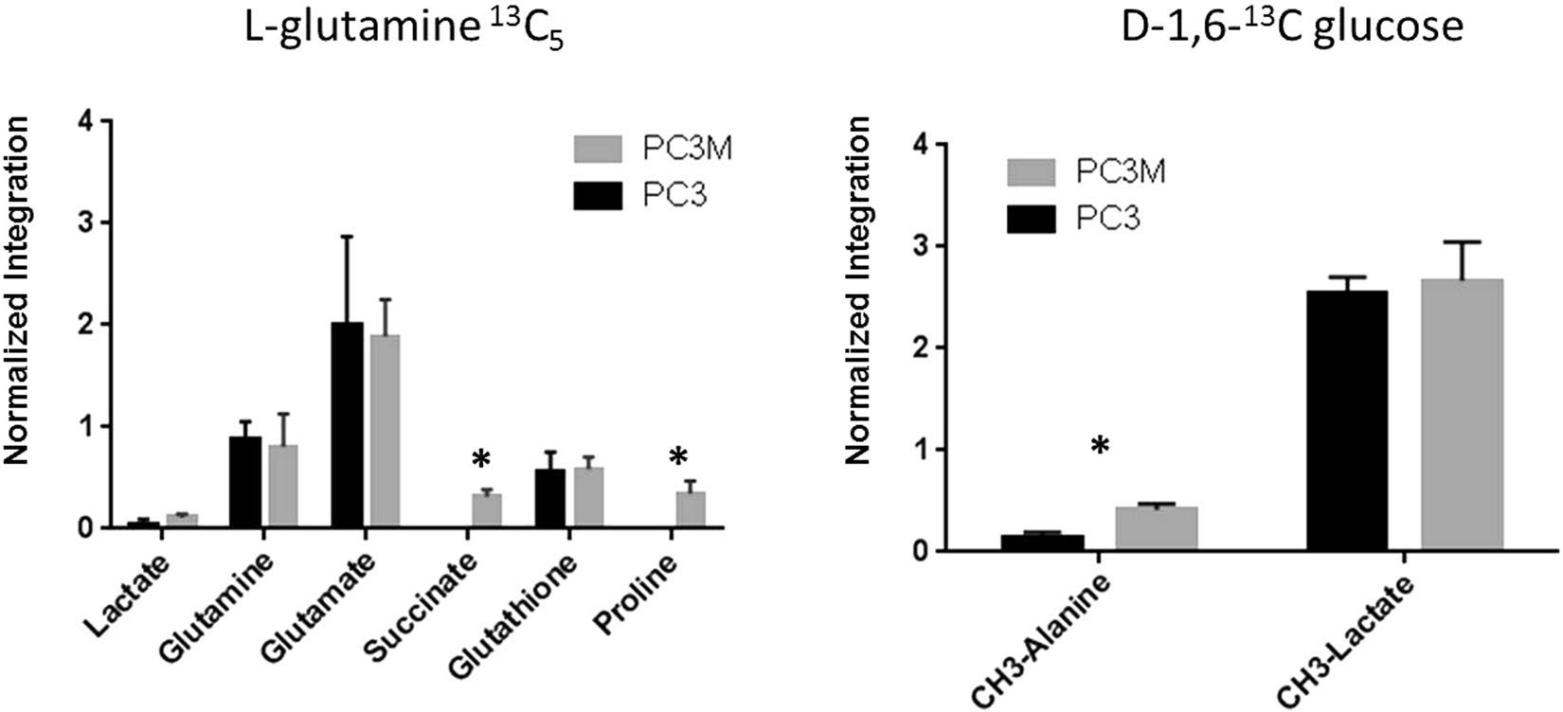
Carbon-13 feeding studies reveal differences in glutamine utilization. ^13^C-labeled feeding studies were done to elucidate glucose and glutamine utilization by PC3 and PC3M cells. Four plates of PC3 and PC3M cells were incubated for 24 hours with 1,6-^13^C glucose or carbon-13 fully labeled glutamine. Metabolites were determined by ^13^C high-resolution spectroscopy. The results show increased carbon-13 label from glutamine in succinate and proline in PC3M cells. Unpaired t-test using Holm-Sidak method was used to determine statistical significance.

To further explore the differences in glutamine and glucose utilization in PC3 and PC3M cells lines, carbon-13 feeding/tracing experiments were performed. Similar lactate values were seen in both cell types and in both tracing experiments; however with the carbon-13 glutamine feeding experiments we selectively found the label in intracellular succinate and proline in the PC3M cell line (Fig. 2). This result, along with our proton spectroscopy results, emphasizes the differences in the utilization of glutamine between PC3 and PC3M.

### Hyperpolarized pyruvate studies reveal increased glycolytic flux in PC3 tumors

**Figure 3:**
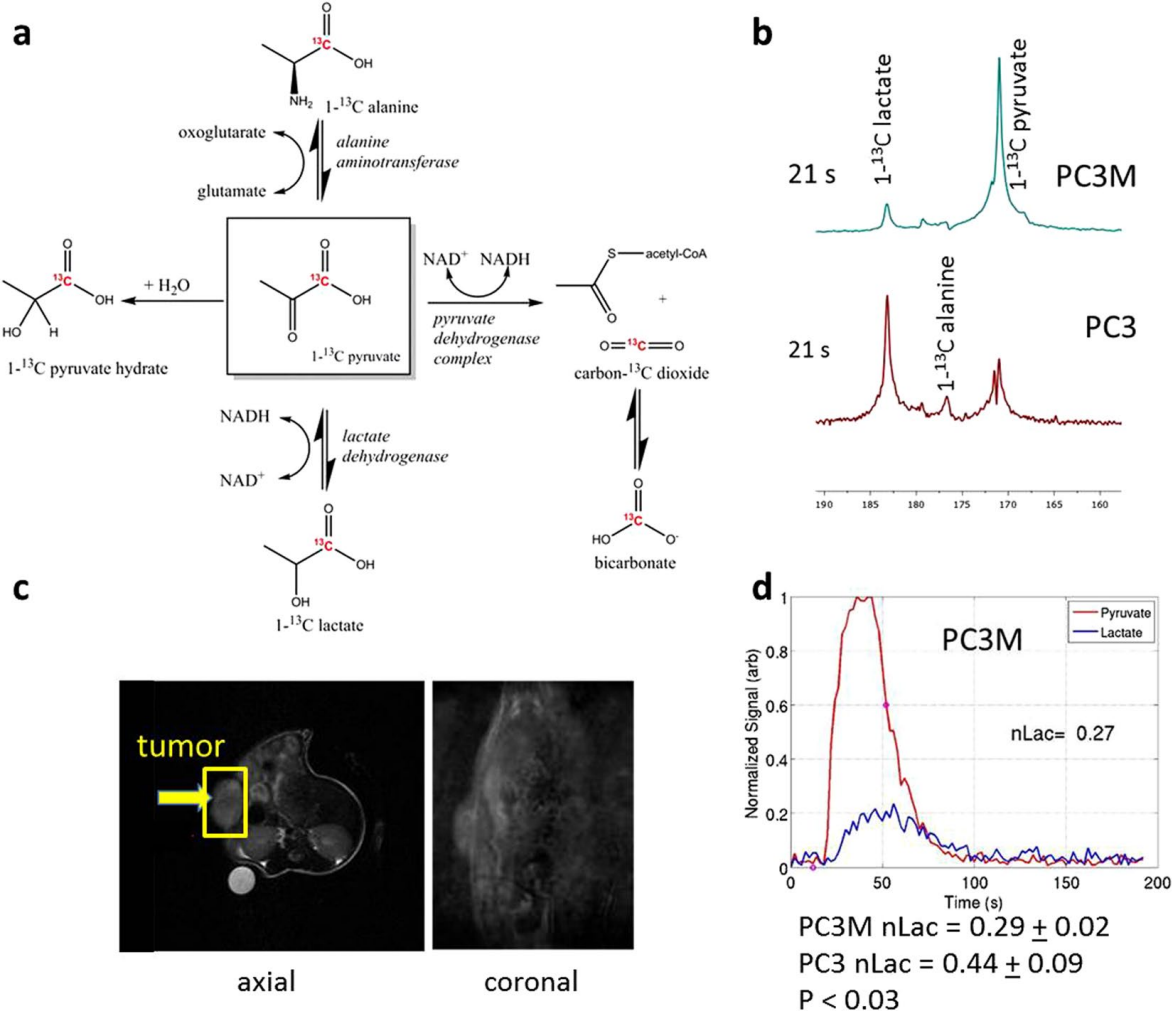
Increased glycolysis observed in PC3 tumors with hyperpolarized pyruvate. (**a**) The schematic illustrates the different pathways through which hyperpolarized 1-^13^C pyruvate can be metabolized in the cell. Because of the quick loss of polarization, resonances for lactate and alanine are the main metabolites detected *in vivo*. (**b**) Spectra from representative PC3M and PC3 tumor-bearing mice taken 21 s after tail vein injection of hyperpolarized pyruvate. The faster conversion rate in PC3 is readily seen. (**c)** ^1^H image of a representative PC3M tumor-bearing mouse. Slice-selective ^13^C spectroscopy was utilized to capture the ^13^C signal in the tumor tissue after injection of hyperpolarized pyruvate. (**d**) Hyperpolarized pyruvate data were processed to generate dynamic curves characterizing the arrival of hyperpolarized pyruvate and its chemical conversion into lactate. Normalized lactate (nLac), defined as the ratio of total cumulative lactate signal to the total carbon-13 signal, was calculated for each ^13^C scan. Normalized lactate conversion was greater in PC3 tumor-bearing animals (0.44 ± 0.09) than in PC3M tumor-bearing animals (0.29 ± 0.02). The statistical significance of the difference between tumor groups was determined by unpaired two-tailed t-test (P < 0.03).

We utilized hyperpolarized 1-^13^C pyruvate to determine the differences in glycolysis of PC3 and PC3M subcutaneous tumors in nude mice. This conversion rate can be measured in real time and *in vivo* by measuring the integration of the lactate signal over the overall hyperpolarized signal. A series of slice-selective ^13^C spectra were collected immediately after injection of tumor-bearing mice with hyperpolarized 1-^13^C pyruvate to observe the arrival of the compound to the tumor and its conversion to lactate. Pyruvate to lactate conversion was greater in PC3 tumors than in PC3M tumors (0.44 ± 0.09 [n = 5] versus 0.29 ± 0.02 [n = 3], P < 0.03) (Fig. 3). This finding corresponds with the greater extracellular lactate production over 24 hours by PC3 cells than by PC3M cells in culture.

### Drug inhibition assays reveal glutamine utilization variability

**Figure 4:**
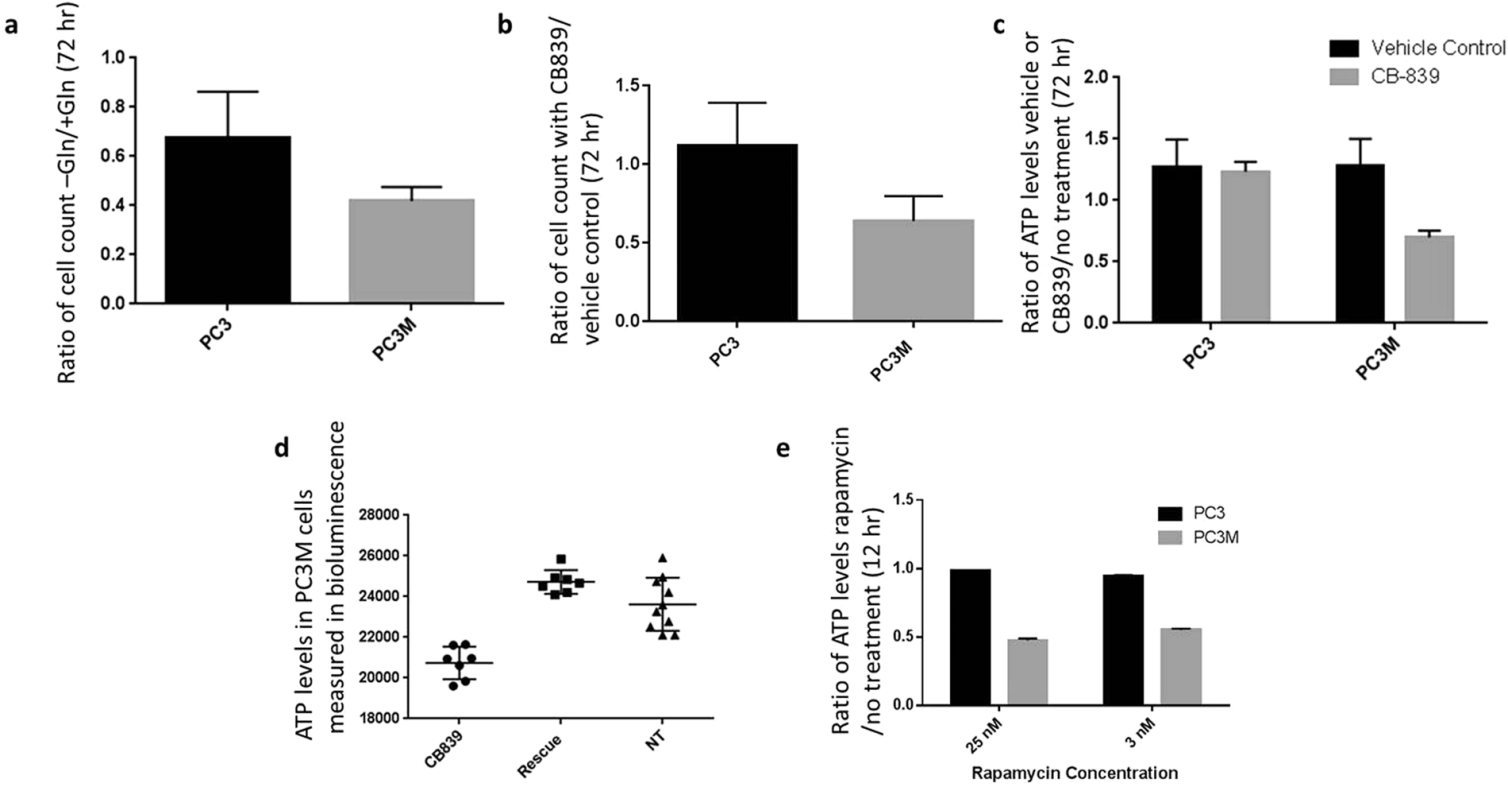
Therapeutic vulnerabilities of PC3M cell line observed in drug assays. PC3 and PC3M cells were treated with various metabolites or inhibitors and the effects on cell viability and proliferation were assessed. Unless otherwise noted, the statistical significance of differences between groups was determined by unpaired two-tailed t-test. (**a**) Comparison of PC3 and PC3M cell counts after 72 hours of growth in the presence or absence of 2 mM glutamine (Gln). (**b**) Comparison of PC3 and PC3M cell counts after 72 hours of treatment with GLS1 inhibitor CB-839 or vehicle. Five to six replicates were used for each condition. In both experiments (**a** and **b**), PC3M proliferation was inhibited to a greater extent than PC3 proliferation (P < 0.03 [+/− Gln], P < 0.00001 [+/− CB-839]). PC3 proliferation was not inhibited by CB-839 but was inhibited by complete exclusion of glutamine in the media. Both cell lines proliferated in the absence of glutamine and in the presence of CB-839. (**c**) ATP levels in cells treated for 72 hours with vehicle or with 1 μM CB-839 over untreated control. Five to six replicates were used for each condition. Levels of ATP were reduced only in the treated PC3M samples (P < 0.0001). (**d)**. ATP levels were equivalent to those in cells treated with vehicle when cells treated with 1 μM CB-839 were also incubated with 4 mM dimethyl 2-oxoglutarate, showing that DMKG rescued the citric acid cycle. (**e**) Treatment with mTOR inhibitor rapamycin (3 nM or 25 nM) significantly reduced ATP levels in PC3M cells compared to vehicle control but did not reduce ATP levels in PC3 cells. The statistical significance of the differences was determined by two-way ANOVA (P < 0.00001, seven replicate samples per condition).

PC3 and PC3M cells were grown with and without added glutamine (Fig. 4a) or in the presence of 0.1% DMSO (vehicle control) or 1 μM of GLS1 inhibitor CB-839 for 72 hours (Fig. 4b). Cells were counted after the 72-hour period. Both treatments reduced proliferation of the PC3M cells. Interestingly, both cell lines proliferated without glutamine. Cell viability measurements were performed in both cell lines after incubation for 72 hours with vehicle control or 1 μM CB-839. Levels of ATP were lower in the PC3M cells incubated with CB-839 (Fig. 4c). The ATP level could be rescued by adding 4 mM dimethyl 2-oxoglutarate to the media (Fig. 4d). Dimethyl 2-oxoglutarate is a hydrophobic analog of α-ketoglutarate and has been shown to be metabolized in the citric acid cycle^39,41^. In cells treated with mTOR inhibitor rapamycin (3 nM or 25 nM) for 12 hours, the ratios of ATP levels in treated and control cells showed reduced ATP in PC3M cells but not in PC3 cells (Fig. 4e). These results confirm that glutamine dependence is higher in the metastatic subline PC3M than in PC3 cells and that glutamine is being utilized to fuel the citric acid cycle. These results also suggest that mTOR activation is more prevalent in the PC3M cells than in the PC3 cells.

### Protein levels indicate differences in signaling and glutamine metabolism pathways

**Figure 5:**
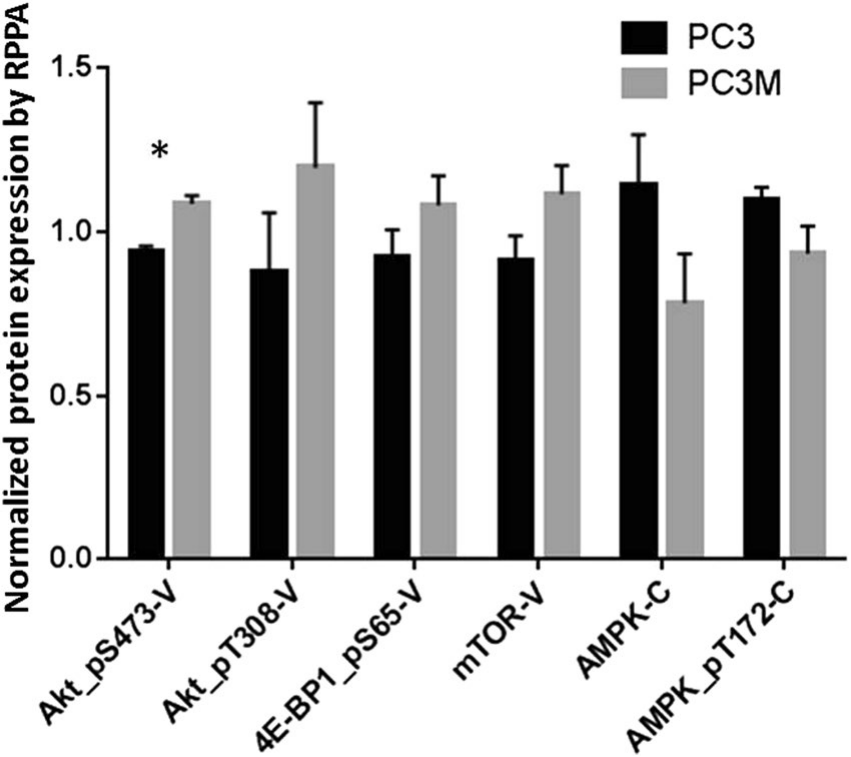
Signaling pathway variation between PC3 and PC3M cells. Reverse-phase protein array (RPPA) analysis was used to identify differences in expression of various proteins in PC3 and PC3M cells (n = 3 samples per cell type). The x-axis labels represent selected proteins targeted by the antibodies used in the analysis; V is validated and C is course. The validated antibodies have been shown in multiple assays to target only the protein named and not to have off-target binding; course antibodies have off-target binding. PC3M cell lysates expressed higher levels of phosphorylated AKT (Akt-pS473, P < 0.01, unpaired t-test using Holm-Sidak method for multiple comparisons), 4E-BP1, and mTOR than PC3 cell lysates. Expression of AMPK and p-AMPK was higher in PC3 cell lysates.

Reverse-phase protein array (RPPA) technology^46^ was used to analyze the cellular protein activity in the lysates from PC3 and PC3M cells (n = 3). Figure 5 highlights a few of the proteins whose levels varied by >0.1 between the cell lines and specifically in the signaling pathway of AKT and AMPK. Levels of phosphorylated S473-AKT (Akt-pS473, P < 0.01, unpaired t-test using Holm-Sidak method for multiple comparisons) was higher in the PC3M cell lysates than in the PC3 cell lysates, while total AKT levels were equal. Levels of p-AMPK were higher in the PC3 lysates than in the PC3M lysates. Both AMPK and AKT are master regulators of cell growth and cellular metabolism. The deviation between the levels of these two proteins could be the underlying cause of the metabolic variability between the two lines.

**Figure 6:**
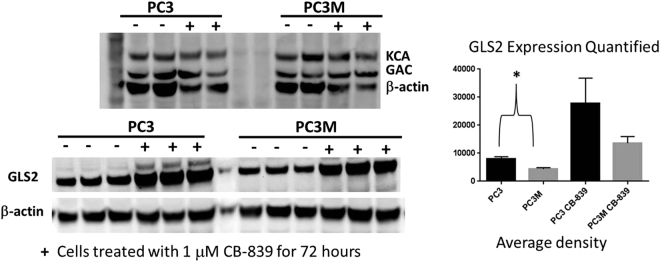
Glutaminase protein expression in PC3 and PC3M cell lines before and after CB-839 treatment. PC3 and PC3M cells were treated (+) or not treated (−) with GLS1 inhibitor CB-839 (1 μM) and their expression of GLS1 and GLS2 proteins were determined by Western blot. Antibodies specific for GAC and KCA were utilized on two replicate samples, while GLS2 expression was determined on three replicates. PC3 cell lysates had higher levels of expression of GLS2 than PC3M cell lysates regardless of CB-839 treatment. The average density of bands in the GLS2 blots were determined by using ImageJ software. The statistical significance was determined by unpaired two-tailed t-test. The difference in GLS2 expression was significant in the untreated cells (P < 0.03) but because of high variability the difference was not significant in the drug-treated cells. Levels of GLS1 (GAC and KGA) were similar in all cell lysates. The full gels can be seen in the supplemental data.

The levels of GLS1 (GAC and KCA) and GLS2 were determined by Western blot in PC3 and PC3M cell lysates treated or not treated with 1 μM CB-839 (Fig. 6). Expression of GLS2 was higher in PC3 lysates than in PC3M lysates in both treated and untreated samples. Levels of GLS1 were approximately equivalent in both cell lines. GLS2 overexpression is observed in both PC3 and PC3M cells with drug incubation indicating that GLS2 could be compensating for GLS1 inhibition. GLS2 overexpression after drug treatment is the highest in PC3 cells. Reduced GLS2 levels with and without drug present may lead to the greater susceptibility of PC3M to CB-839 treatment.

## Discussion

Our results illustrate the metabolic reprogramming that can occur between similar cell lines. Our metabolic results are intriguing because the PC3M line was derived by isolating and growing a lung metastasis of PC3 in a nude mouse^47^. The PC3M line is more metastatic and aggressive than the initial parent cell line^47^. Both lines are androgen receptor–null and therefore castration resistant. Glycolysis was greater in PC3 cells than in PC3M cells both *in vivo* and *in vitro* as evaluated by metabolic imaging and metabolic profiling. Furthermore, variations in ^13^C-labeled glutamine uptake and metabolic products in cultured PC3M cells compared to cultured PC3 cells indicate that glutamine is being utilized to a greater extent to feed the citric acid cycle (succinate labeling) in PC3M cells. In addition, higher levels of succinate and aspartate (both metabolites formed from the citric acid cycle) were observed in PC3M tumor tissue. The level of GLS2 expression was lower in PC3M cells than in PC3. Together, these differences lead to the greater vulnerability of PC3M cells to GLS1 inhibitor CB-839. To our knowledge, this is the first study showing that CB-839 reduces cell proliferation and viability in an isogenic PCa line.

This increase in glutamine utilization in more aggressively metastatic cell lines has been observed also in ovarian cancer^48^, suggesting that glutamine utilization might be directly related to metastatic progression^48^. Jiang *et al*. reported that cells in culture require glutamine reductive decarboxylation to form spheroid structures from anchorage-dependent growth^49^. CB-839 could have a larger effect on metastatic progression than on cell proliferation. Our results reveal that both PC3 and PC3M proliferate even in the complete absence of glutamine, illustrating the dynamic nature of metabolism and the capacity for multiple compounds to be utilized as carbon and nitrogen sources to provide the building blocks for replication.

Our RPPA data revealed differences in levels of phosphorylated AKT (pS473P) and mTOR in PC3M versus PC3 lysates. Incubation with 3 nM rapamycin (an mTOR inhibitor) or 1 μM CB-839 reduced ATP production only in PC3M cells. Phosphorylated AKT is known to activate mTOR, which can stimulate glutamine utilization^50^, and increases in glutamine concentration can directly modulate the mTOR pathway^51^. In future studies, we will determine whether regulation of this pathway can further explain the differences in glutamine utilization between these two cell lines.

In conclusion, we observed greater glycolytic flux in PC3 tumors than in PC3M tumors in mice and see increased glutamine utilization in the PC3M cell line. Once we understand the mechanisms underlying this metabolic variation between PCa subtypes, we may be able to use hyperpolarized metabolic imaging to provide a rationale for individualized combinations of metabolic pathway inhibitors and chemotherapy and potentially improve the outcome of prostate cancer patients.

## Materials and Methods

### Cell lines and culture conditions

Four human PCa lines were used: RWPE-1 (non-tumorigenic, considered benign), RWPE-2 (non-metastatic), PC3 (aggressive, castration resistant), and the aggressive subline PC3M (castration resistant)^45^. RWPE1, RWPE2, and PC3 cells were purchased from ATCC. PC3M cells were purchased from the MD Anderson cell line core facility, which validates all of the cell lines it provides. Cell lines were tested for mycoplasma every couple of months by MD Anderson cell line core facility. For intracellular metabolite analyses, RWPE1 and RWPE2 cells were cultured in keratinocyte serum-free media supplemented with bovine pituitary extract and recombinant human epidermal growth factor. PC3 cells were cultured in F12-K media supplemented with 10% fetal bovine serum (FBS), and PC3M cells were cultured in RPMI1640 media supplemented with 0.1 mM modified essential media (MEM) and 10% FBS. All media were supplemented with 10 U/mL penicillin and 10 µg/mL streptomycin. For extracellular metabolite determination, PC3 and PC3M cells were seeded in optimized media and, after 24 hours, exchanged into RWPE1/2 media. All media were purchased from Gibco and Corning.

For CB-839 studies and glutamine studies, PC3 and PC3M cells were grown in RPMI1640 media (with or without glucose/glutamine) supplemented with 10% FBS, 10 U/mL penicillin, and 10 µg/mL streptomycin. The media was supplemented with frozen aliquots of glutamine and glucose prior to experiments.

### Mouse models

All experimental methods involving mice were performed in accordance with the guidelines and regulations of the MD Anderson Institutional Animal Care and Use Committee (IACUC), and the animal protocols used were approved by that committee. Male nude mice >5weeks old were each injected with ~5 × 10^6^ cells subcutaneously on the rear flank. When the resulting tumors had grown to approximately 1 to 1.3 cm in size, the animals underwent imaging as described in a later subsection. The animals were sacrificed with isoflurane overdose and cervical dislocation 6–48 hours after imaging, and tumor tissues were removed and flash-frozen for metabolic studies.

### High-resolution magnetic resonance spectroscopy

Magnetic resonance spectroscopy (MRS) was used for all metabolic analyses. MRS allows chemical resonances of metabolites to be determined in one experiment. For all high-resolution MRS, one-dimensional ^1^H proton spectroscopy was performed with water suppression on a 500 MHz Bruker Biospin Avance III high-definition nuclear magnetic resonance (NMR) instrument equipped with a Prodigy BBO cryoprobe. The cryoprobe increases the sensitivity of the measurement 3- to 4-fold. All supplies (deuterium oxide [D_2_O], 3-(trimethylsilyl)-1-propanesulfonic acid-d_6_ sodium salt [DSS-d_6_], and potassium phosphate buffer [K_2_HPO_4_, pH 7.4]) were purchased from Sigma-Aldrich and used without further purification.

To determine intracellular metabolite levels, cells (0.5–3.0 × 10^7^ per sample) were trypsinized and pelleted at approximately 80% confluence, and metabolites were extracted using an ice-cooled 2:1 methanol to water solution (3 mL) and MP Biomedicals lysing matrix D beads (~500 µL per 10^7^ cells). The homogenates were then subjected to centrifugation for 10 min at 4000 *g*, the supernatant removed and lyophilized overnight, and the remaining metabolites dissolved in D_2_O with 0.5 mM DSS-d_6_ and 50 mM K_2_HPO_4_. The one-dimensional ^1^H-NMR spectra used 256 scans and a spectral width (SW) of 10245 Hz and was referenced to DSS at 0.00 ppm. Water suppression was performed with presaturation. For the metabolites assigned and quantified by using ^1^H-NMR spectroscopy, four or five replicates of each cell type were analyzed. Data were processed/analyzed with Chenomx (Chenomx, Inc.), MestreNova (Mestrelab Research), and/or Topspin (Bruker Biospin) software. Integrated values of intracellular metabolites were determined by taking the ratio of the resonance for each metabolite over the DSS-d_6_ peak to the total integration of all resonances in the spectra. This allowed normalization of the probe performance within each sample. Metabolite resonances were identified through reference to either of two online metabolomics databases, Human Metabolome Database (http://www.hmdb.ca)^52^ or Biological Magnetic Resonance Bank (http://www.bmrb.wisc.edu/metabolomics) and, when necessary, confirmed by spiking the sample with a known amount of the metabolite in question. To easily discern the metabolic profile of the more aggressive PCa lines, the fold difference of each metabolite to RWPE1 (indolent PCa) was graphed (Fig. 1c).

To determine the differential uptake and excretion of some of the dominant media metabolites, five plates (145 × 20 mm) of each cell line were cultured so that there were five replicates at each time point. Because of the large difference in cell size, samples were not normalized to cell count; instead, cell cultures were carefully monitored to allow initiation of the media time course experiment when cells had reached 80% confluence. When cell cultures became approximately 80% confluent, uptake and excretion of metabolites were monitored in the four cell lines over a period of 24 hours in keratinocyte serum-free media. Aliquots of media were taken at the beginning (t = 0) and end of the 24-hour period (t = 24) from five separate tissue culture plates for each cell line. Figure 1b plots the fold change in the integrated value of each metabolite over a 24 hour period.

To determine intracellular metabolite values from subcutaneous tumors, flash frozen tissues (100 mg per sample) were homogenized using a liquid nitrogen–cooled mortar and pestle; metabolites were extracted by a method similar to that used for cell pellets. Integrated values for each metabolite were determined by taking the ratio of the resonance for each metabolite over the DSS-d_6_ peak to the total integration of all resonances in the spectra. To visualize these differences easier, each integration value was multiplied by a scaling factor to account for the number of protons each chemical resonance corresponded to (1000 (1H), 500 (2H), 250 (4H), Fig. 1d).

### Carbon-13 tracing experiments

PC3 and PC3M cells were cultured for 24 hours in glucose/glutamine–free Dulbecco MEM to which either fully labeled ^13^C-glutamine (final concentration 3 mM) or 1,6-^13^C glucose (final concentration 10 mM) was added (Fig. 2). After the 24-hour period, the media was removed, and the cells were washed twice with phosphate-buffered saline solution (PBS), trypsinized, pelleted, and then homogenized by the same method as for non-tracing experiments. Labeled compounds (Sigma-Aldrich) were used without any further purification. Lyophilized metabolites were redissolved in 50 mM phosphate buffer (pH 7.4) with 5 mM DSS-d_6_. Proton decoupled one-dimensional ^13^C spectroscopy was run (4096 averages, relaxation delay 6s, SW 29760 Hz, 30° flip angle) on each sample. Two-dimensional heteronuclear single-quantum correlation experiments were run on each sample to confirm the assignment of each metabolite (16 averages, relaxation delay 3s, 256 points in 2^nd^ dimension, SW 6500 Hz for ^1^H, SW 20800 Hz for ^13^C).

### Protein array and Western blotting analyses

For reverse-phase protein array (RPPA) analysis, flash-frozen cell pellets comprising 5 × 10^6^ cells were transferred to the MD Anderson RPPA core facility for processing and analysis. The pellets were subjected to lysis using RPPA lysis buffer, and lysates were serially diluted manually with five 2-fold dilutions of lysis buffer and then printed on nitrocellulose-coated slides^53^. The NormLinear algorithm, which corrects for protein loading and antibody variations, was used to determine the difference in protein expression between groups of lysates^54,55^.

For detection of specific proteins via Western blotting, PC3 and PC3M cells were grown in complete RPMI1640 media supplemented with 10% FBS, 10 U/mL penicillin, and 10 µg/mL streptomycin in 6-well plates. When cells became 60% confluent, fresh media was added with or without GLS1 inhibitor CB-839 (1 μM). After 72 hours, the media was removed and the cells were detached using trypsin, counted, and pelleted. Whole-cell lysates were prepared by using RIPA buffer (Thermo Scientific), and total protein concentration was determined by the Pierce BCA protein assay (Thermo Scientific). Protein lysates were denatured by boiling in sodium dodecyl sulfate (SDS) sample buffer for 5 min at 95 °C and then loaded onto a 4–12% mini-precast polyacrylamide gel (Nupage). Membranes were blocked with Odyssey blocking buffer for 60 minutes at room temperature, and then were incubated with antibodies recognizing KCA (20170-AB, ProteinTech)^39^, GAC (199581-AP, ProteinTech)^39^, or GLS2 (NBP1-76544, Novus Biologicals) overnight at 4 °C and with β-actin (SC477778, Santa Cruz Biotechnology) for 1 hour at room temperature. Molecular weights were determined using SeeBlue Plus2 Pre-stained protein standard (LC5925, Invitrogen). Secondary antibodies conjugated to infrared (IR) fluorescent dyes were utilized to image bands using the LI-COR Odyssey IR fluorescent system. Densitometry analysis was performed using ImageJ software (Fig. 6, Supplemental Figure).

### Imaging procedures and hyperpolarized pyruvate

Ox063 trityl radical (Oxford Instruments) was mixed with neat 1-^13^C pyruvic acid (Sigma-Aldrich) to a concentration of 15 mM. Aliquots of this solution (20 μL) along with 0.4 μL of 50 mM Gd^3+^ relaxation agent (Magnevist, Bayer Healthcare) was loaded into a commercial HyperSense dynamic nuclear polarization (DNP) polarizer (Oxford Instruments) and irradiated at a microwave frequency of 94.100 GHz for 30–40 minutes (until the polarization plateau was reached) and then dissolved in 4 mL buffer solution containing 40 mM Tris (7.6 pH preset), 80 mM NaOH, 0.1 g/L EDTA, and 50 mM NaCl.

For imaging, the tumor-bearing mice were anesthetized with 3% isoflurane mixed with oxygen and then maintained with 0.5–1% isoflurane. Animals were placed on a heated pad and their respiration and heart rate monitored during imaging procedures. The neutral (pH 7-8) 80 mM hyperpolarized 1-^13^C pyruvate solution was injected into each mouse via tail vein catheter.

All imaging and spectroscopy were performed with a dual tuned ^1^H/^13^C volume coil (Doty Scientific) or a ^1^H/^13^C volume coil (Bruker BioSpin) in a 7T Bruker Biospec horizontal bore MR scanner equipped with a single channel for carbon excitation/reception. Proton anatomic images were taken using a multi-slice T2-weighted RARE sequence. A small 8 M ^13^C-urea phantom doped with gadolinium-DPTA was next to the tumor for chemical shift referencing. A series of slice-selective ^13^C spectra (field-of-view 40 × 40 mm, slice-thickness 8–12 mm) were collected immediately after injection of hyperpolarized 1-^13^C pyruvate. The single slice was placed over the tumor using the multi-slice proton imaging sequence for placement. A total of 90 transients were acquired with a time delay of 2 seconds between each transient (total time 3 minutes). Each transient utilized a 15–20° degree flip angle excitation pulse (gauss pulse) and 2048 data points. Metabolic flux ratios of pyruvate to lactate were determined with a unidirectional model (Fig. 3). Data were processed both in MATLAB (MathWorks Inc) or MestReNova. The dynamic spectra were manually phased and line-broadening was applied (10 to 15 Hz). The area under the spectral peaks for pyruvate and lactate were integrated over the whole array. Normalized lactate (nLac) ratio was calculated as lactate over the sum of pyruvate and lactate signals^56^.

### CB-839, glutamine, and rapamycin cell proliferation and viability assays

For cell viability assays, PC3 or PC3M cells (2,000) in RPMI media were added to wells of a 96-well plate. GLS1 inhibitor CB-839 was dissolved in dimethyl sulfoxide (DMSO) at a concentration of 10 mM. We prepared 100 μl aliquots of the solution which were kept frozen at −20 °C. For each experiment, a separate frozen aliquot of the 10 mM CB-839 solution was utilized to generate our experimental 1 μM CB-839 conditions and any remaining material was disposed of. The plates were incubated in the CO_2_ incubator for 72 hours. Vehicle control wells were treated with 0.1% DMSO. The cells were then subjected to the Promega CellTiter-Glo Luminescent Cell Viability Assay (G7571). A similar assay was utilized to determine the effect of mTOR inhibitor rapamycin, except cells were seeded at 5000 cells per well at the beginning of the 12-hour incubation.

For the cell proliferation assay, PC3 or PC3M in RPMI media were added to each well of a 24-well plate (5000 cells per well). Cells were treated with CB-839 (1 μM) or 0.1% DMSO. Plates were incubated in a CO_2_ incubator for 72 hours. Cells were then trypsinized and counted using a Bio-Rad TC20 Cell Counter. In another set of experiments using the same parameters, cells were grown in the presence or absence of glutamine and counted after 72 hours (Fig. 4).

### Statistical analyses

All statistical analyses were performed with GraphPad Prism version 6.00 for Windows, GraphPad Software, La Jolla California USA, www.graphpad.com. P values are given for all analyses and statistical significance determined to be any P value below 0.05. All experimental data are shown as mean ±SEM.

### Data availability

The datasets generated during and/or analyzed during the current study are available from the corresponding author on reasonable request.

## Electronic Supplementary Material

Supplementary Information
