# Supplementary material for: Metabolic Differences in Glutamine Utilization Lead to Metabolic Vulnerabilities in Prostate Cancer
Source: Sci Rep. 2017 Nov 23;7:16159. doi: 10.1038/s41598-017-16327-z (PMC5701017; doi:10.1038/s41598-017-16327-z)

**Metabolic Differences in Glutamine Utilization Lead to Metabolic Vulnerabilities in Prostate Cancer (Supplement)**

Niki Marie Zacharias^1,2^, Christopher McCullough^3‡^, Sriram Shanmugavelandy^1‡^, Jaehyuk Lee^1^, Youngbok Lee^4^, Prasanta Dutta^1^, James McHenry^1^, Linda Nguyen^1^, William Norton^5^, Lawrence W Jones^6^, Pratip Bhattacharya^1^

^1^Department of Cancer Systems Imaging, The University of Texas MD Anderson Cancer Center, Houston, TX;

^2^Department of Bioengineering, Rice University, Houston, TX;

^3^Institute for Bioscience and Biotechnology Research, National Institute of Standards and Technology, Rockville, MD;

^4^Department of Bionano Technology. Hanyang University, ERICA campus, Ansan, Korea;

^5^Department of Veterinary Medicine, The University of Texas MD Anderson Cancer Center, Houston, TX;

^6^Huntington Medical Research Institutes, Pasadena, CA

^‡^These two authors contributed equally.

The full blots for the GLS1 and GLS2 (Figure 6) are below. Cells were exposed to 1 μM CB-839 for 72 hours (treated).

Western blots:

Supplemental Figure 1: GLS1 in PC3 and PC3M cells treated with CB-839.


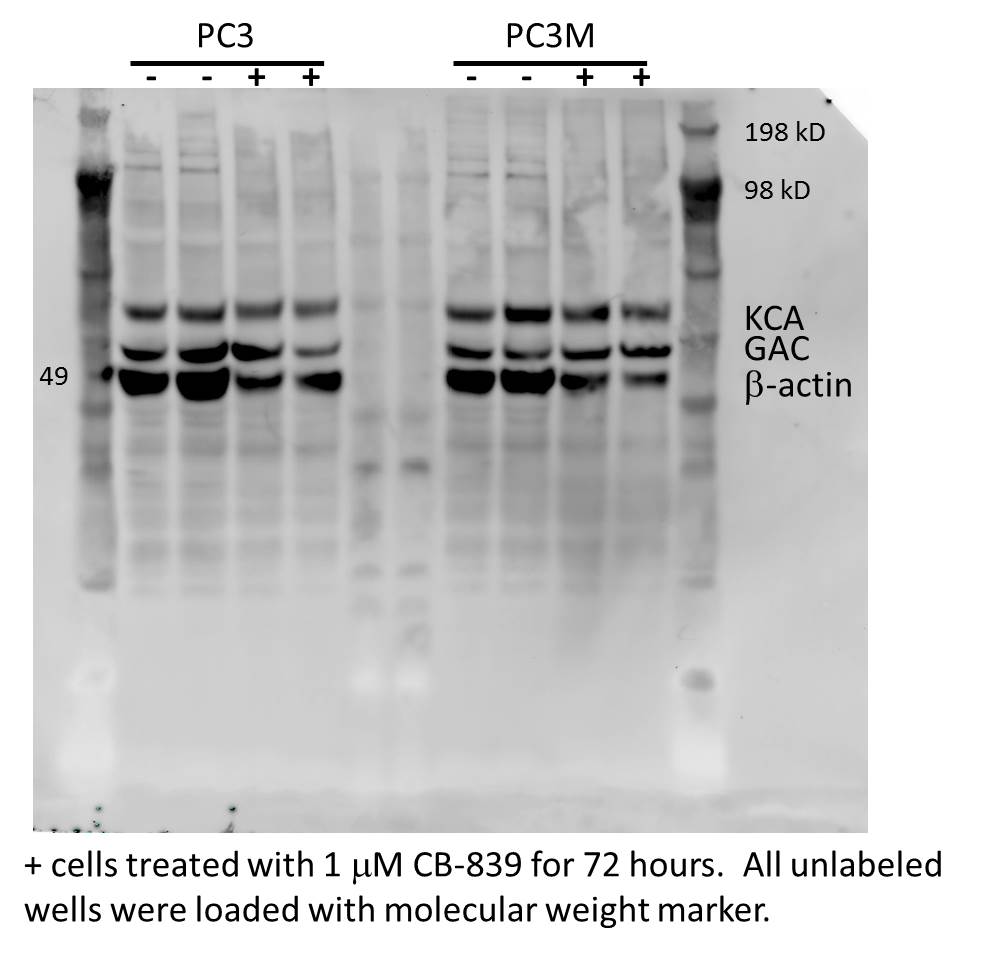


Supplemental Figure 2: GLS2 in PC3 and PC3M cells treated with CB-839


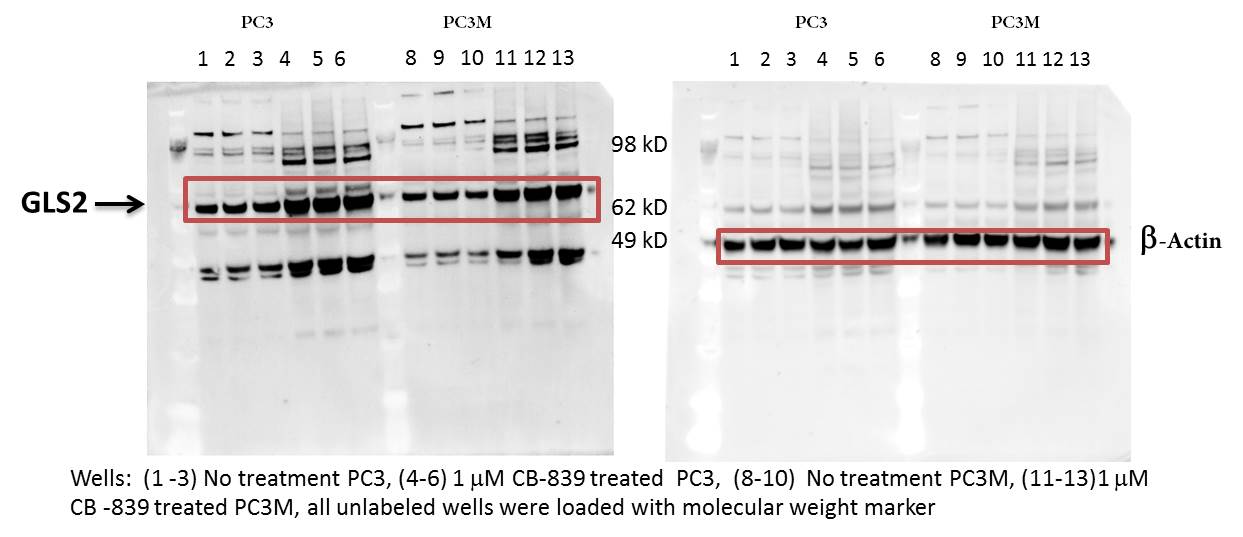

Supplement: Supplementary file 1 — Supplementary Information [file 41598_2017_16327_MOESM1_ESM.doc]
